# Multivariate multiscale entropy (*mMSE*) as a tool for understanding the resting-state EEG signal dynamics: the spatial distribution and sex/gender-related differences

**DOI:** 10.1186/s12993-023-00218-7

**Published:** 2023-10-05

**Authors:** Monika Lewandowska, Krzysztof Tołpa, Jacek Rogala, Tomasz Piotrowski, Joanna Dreszer

**Affiliations:** 1 Department of Clinical Psychology and Neuropsychology, Institute of Psychology, Faculty of Philosophy and Social Sciences, Nicolaus Copernicus University in Torun, Gagarina 39 Street, 87-100 Torun, Poland; 2 Faculty of Physics, University of Warsaw, Pasteur 5 Street, 02-093 Warsaw, Poland; 3 Institute of Engineering and Technology, Faculty of Physics, Astronomy and Informatics, Nicolaus Copernicus University in Torun, Grudziądzka 5 Street, 87-100 Torun, Poland

**Keywords:** Resting-state networks, EEG, Multivariate multiscale entropy, EEG complexity, EEG dynamics

## Abstract

**Background:**

The study aimed to determine how the resting-state EEG (rsEEG) complexity changes both over time and space (channels). The complexity of rsEEG and its sex/gender differences were examined using the multivariate Multiscale Entropy (*mMSE*) in 95 healthy adults. Following the probability maps (Giacometti et al. in J Neurosci Methods 229:84–96, 2014), channel sets have been identified that correspond to the functional networks. For each channel set the area under curve (AUC), which represents the total complexity, *MaxSlope*—the maximum complexity change of the EEG signal at thefine scales (1:4 timescales), and *AvgEnt*—to the average entropy level at coarse-grained scales (9:12 timescales), respectively, were extracted. To check dynamic changes between the entropy level at the fine and coarse-grained scales, the difference in *mMSE* between the #9 and #4 timescale (*DiffEnt*) was also calculated.

**Results:**

We found the highest AUC for the channel sets corresponding to the somatomotor (SMN), dorsolateral network (DAN) and default mode (DMN) whereas the visual network (VN), limbic (LN), and frontoparietal (FPN) network showed the lowest AUC. The largest *MaxSlope* were in the SMN, DMN, ventral attention network (VAN), LN and FPN, and the smallest in the VN. The SMN and DAN were characterized by the highest and the LN, FPN, and VN by the lowest *AvgEnt.* The most stable entropy were for the DAN and VN while the LN showed the greatest drop of entropy at the coarse scales. Women, compared to men, showed higher *MaxSlop*e and *DiffEnt* but lower *AvgEnt* in all channel sets.

**Conclusions:**

Novel results of the present study are: (1) an identification of the *mMSE* features that capture entropy at the fine and coarse timescales in the channel sets corresponding to the main resting-state networks; (2) the sex/gender differences in these features.

**Supplementary Information:**

The online version contains supplementary material available at 10.1186/s12993-023-00218-7.

## Introduction

Despite an increasing number of studies [6, 40, 73, 95], the resting-state complexity of EEG signals remains poorly understood. New complexity measures, which are not directly comparable, along with unstructured terminology, complicate this task. We analyzed the bioelectrical resting-state signal complexity with the use of multivariate extension of Multiscale Sample Entropy (*MSE* [15]; multivariate extension of *MSE*, [2–4]. Since this method estimates the repetition in the temporal patterns of a signal across multiple timescales and space (electrodes) (spatiotemporal complexity profiles of signals [15]), it appears to be the best statistical approximation of the system dynamics and does not directly represent information processing but rather the conditions in which it occurs.

Complexity understood in this way may reflect the functional couplings (temporal correlations) between anatomically distinct regions [59, 66, 94, 97], the range of possible responses to environmental demands [35], implementation of Bayesian inference by the neural circuits [57], the brain’s ability to explore alternative states [23, 34, 43, 63], a moderate noise level in the system which aids information processing [31], regulation of synchrony between/within areas [37] or the interplay between functional segregation and integration within the brain [86, 92]. The brain signal complexity can be best explored at rest when diverse states (functional networks) are activated or deactivated over time, the brain is free from tasks and stimuli influence, and never settles on a fixed point [13, 22]. The EEG/MEG techniques, with a millisecond temporal resolution, are considered to be the most adequate to detect these short and fast network state transitions [53, 84].

The EEG/MEG signals could be treated as external observations of a dynamical system at rest [82, 87]. To investigate this system we propose to use the sample entropy (*SampEn*) measure, introduced specifically for the analysis of non-stationary, physiological signals [76]. The *SampEn* is an unbiased version of the approximate entropy (*ApEn*), which essentially measures the logarithmic likelihood that, for *n* points of time series data, vectors of length *m* that are within radius *r* from each other, remain within this radius from each other if their length is incremented to *n* + 1 data points [70]. From a mathematical perspective such formulation represents a real-world computable analogue of the Kolmogorov-Sinai entropy (*KS* entropy) of a dynamical system which is obtained as a theoretical value from the same expression as used for *ApEn*, in the limit of data points *n* and vector length (i.e., the embedding dimension) *m* approaching infinity and the similarity radius approaching zero [30, 70]. Therefore, for the EEG/MEG signals, *ApEn* and its unbiased extension* (SampEn)* allow to capture neural network state transitions by evaluating their complexity (entropy) in the time domain, as both can be viewed as computable approximations of the *KS* entropy of the dynamical system of a working brain. Utilizing this fact, Costa, Goldberger and Peng (2002) proposed the Multiscale Sample Entropy (*MSE*) measure by computing the *SampEn* over coarse-grained time series obtained from the original signal by averaging it over non-overlapping segments of increasing length (scale). Computation of the *SampEn* over subsequent scales allows to distinguish between complexity profiles (understood as values of *SampEn* across scales) of signals conveying information only on shortest (non-averaged) scales such as white noise, and complexity profiles of signals such as colored noise, which exhibit more complex information (i.e., higher entropy represented by *SampEn* values) on longer (averaged) scales.

Building on the *MSE* approach, for a multivariate signal such as EEG, the multivariate *MSE* (*mMSE*), proposed by Ahmed and Mandic [2] and [3, 4], examines the complexity both across time (scales) and space (electrodes) and is able to reveal spatiotemporal complexity profiles of multivariate signals, as opposed to only temporal complexity profiles obtained through univariate *MSE* method. As the EEG/MEG signals are multivariate, the *mMSE* method has been selected as the one providing the most insight into the entropy of the dynamical system of a working brain through its spatiotemporal complexity profiles.

The resting-state EEG (rsEEG) studies have produced inconsistent results regarding the scalp topography of entropy parameters. For example, the maximal complexity values were located over the posterior region [6, 40] or the frontal, central and temporal areas [95]. Racz et al. [73] found the highest permutation entropy measures of rsEEG activity at the electrodes corresponding to the somatomotor and dorsal attention networks. In our previous study [29] the *mMSE* method revealed the largest complexity of the signals from the electrodes placed over the bilateral parietal cortex whereas the smallest entropy values were obtained separately for the left and right frontal and parietal areas.

The current work presents the *mMSE* results of spontaneous EEG signal acquired using the channel sets assigned to the 7 main functional resting-state networks [88], extracted from a large fMRI database following the probability maps provided by Giacometti et al. [38]. Therefore, we expect that our rsEEG entropy outcomes will resemble the resting-state fMRI complexity patterns, found by other authors, i.e. the highest entropy values in the intrinsic (large-scale) neural networks (the default mode network, DMN and/or the frontoparietal network, FPN) [59, 67–69] and the lowest—in the sensory networks (somatomotor, visual, auditory) [47, 65, 67, 69, 99]. The greatest complexity of spontaneous EEG signal over the areas corresponding to the DMN might reflect constant information processing within and across this network [11, 74]. Since the FPN is connected with the DMN for executive control of introspective processes [26] which are favored by a resting condition, a high entropy level in this network is also expected. The sensory networks might be characterized by the lowest rsEEG signal entropy due to a lack of external stimuli or motor reactions to be performed at rest.

We hypothesize that the intrinsic, distributed cognitive control networks (the DMN and the FPN), will demonstrate the smallest complexity of spontaneous EEG signal at the fine scales (considered as reflecting local information processing) and the greatest entropy at the coarse scales (associated with long-range interactions in the brain) [64, 93, 97]. The task-positive networks, on the other hand, as dependent on task demands, might be characterized by the lowest entropy levels at both timescales at rest.

Furthermore, given the limited number of studies showing the dynamics of changes in entropy level across the timescales, we intend to investigate whether a relatively high complexity of each network at the fine scales is maintained or reduced at the coarse scales.

The second objective of the present study was to determine the sex/gender (s/g) differences in the *mMSE* parameters of rsEEG activity. Overall, regardless of the different methods used to calculate neural complexity, women’s brains have been more complex than men’s [1, 32, 50, 56, 72, 98]. For example, Luders et al. [56] found greater right-hemispheric cortical complexity, i.e. spatial frequency of the brain surface gyrification and fissuration, in females compared to males. Wang demonstrated higher *SampEn* values of resting-state fMRI activity in women than men in most cortical areas [98]. Some rsEEG studies also revealed that females, relative to males, displayed greater complexity of the signals recorded over the anterior, central and posterior regions [1, 32, 72].

The s/g differences in rsEEG complexity have been also investigated separately for the frequency bands [46, 50]. Specifically, women exhibited higher *ApEn* parameters than men in the lower bands (delta, theta, low alpha) whereas in the high frequency bands (beta, gamma), men demonstrated greater entropy levels [46]. These outcomes suggest a complex pattern of s/g differences in the excitability of resting-state networks.

In our previous work [29] we found different patterns of relationship between fluid intelligence and the spontaneous EEG signal entropy in men and women. Here we will investigate the ‘pure’ s/g differences in the *mMSE* features, without a reference to any cognitive task. In line with the previous studies [1, 72, 98] we hypothesize that women will demonstrate higher total rsEEG complexity than men and this effect will be more likely observed for all analyzed electrode sets (networks).

At rest women display stronger functional connections within the DMN [5, 10, 20, 77] and spent more time in this network compared to men [19, 90] while men have more interactions in the somatomotor network (SMN) [5, 77] and a greater occurrence rate of microstate corresponding to the SMN [54]. Therefore, considering the neural complexity as reflecting functional connectivity or the brain capacity to explore alternative states, we might assume that, in our study, women will show a higher entropy level of rsEEG signal from the electrodes corresponding to the DMN and men will have greater degrees of entropy in the sensory networks.

Men and women are thought to demonstrate different patterns of local and global information processing in the brain [45, 46, 79, 89, 100]. Assuming that the brain signal complexity at fine scales represents local information processing and the entropy at coarse scales reflects long-range interactions [17], we expect to see s\g differences in the *mMSE* features corresponding to both these scales. Following the resting-state fMRI studies showing that women have more within-network (short-range) functional connections and men produce more (long-range) interactions between the attention, memory, default mode and sensory networks [45, 79, 89], we hypothesize that females will demonstrate a higher entropy level at the fine scales whereas males will display greater complexity at the coarse scales.

## Materials and methods

### Participants

Out of 100 healthy young adults, 95 (42 women and 53 men, mean age = 25.74 ± 4.5 year, age range: 20–41 years, for details refer to Additional file 1: Appendix S1), right-handed, with normal or corrected-to-normal vision, comprised the study sample (5 persons were excluded due to excessive artifacts in the EEG signal). All participants did not suffer from any neurological/psychiatric disorders, had no history of brain injury or drug abuse, and did not take any medications affecting the Central Nervous System (as was revealed by a screening procedure under the supervision of a clinical psychologist and a neurologist). All participants received monetary remuneration for their participation in the study.

The study was approved by the Research Ethics Committee at the Faculty of Humanities, Nicolaus Copernicus University in Toruń, Poland (The Consents No. 6/2018 and 5/2021) and by the Bioethics Committee of the Nicolaus Copernicus University in Toruń, Poland (The Permission No. KB 196/2016). The study is congruent with the principles of the WMA Declaration of Helsinki. Each subject provided written informed consent to take part in the study after all procedures had been fully explained.

### EEG data acquisition and pre-processing

Each participant went through a 5-min session of rsEEG signal acquisition with eyes open. After arrival and before the EEG recording procedure, participants were asked to sit at rest in order to calm down emotions which could potentially affect resting state EEG. The instruction was to focus on the fixation point in the center of the screen and do not think of anything in particular. The data were acquired using 128 electrodes Ag/AgCl electrodes (Actipower and Acticap; Brain Products GmbH) at a sampling rate of 500 Hz. The electrodes were positioned according to the extended 10–20 system. The signal was referenced to FCz and FPz was the ground electrode. The impedance was kept below 10 kΩ during the whole data registration.

The data were processed using MATLAB (ver. R2017a, Mathworks Inc., Natick MA, USA) and the EEGLAB toolbox (ver. 14) [24]. EEG signals were down-sampled to 256 Hz and high pass (> 1 Hz) filtered. Bad channels were removed using an automated procedure (POP_REJCHANSPEC) based on signal SD (rejection threshold of  > 5 SD was used for the frequency range: 0–5 Hz and > 2.5 SD for the frequency range: 5–40 Hz). Epochs containing unusually high amplitudes were detected and removed using a threshold of 444 μV. The remaining signal was low-pass filtered (< 40 Hz) and re-referenced to the average (common) reference. Epochs containing unusually high amplitudes were found and removed using a threshold of 222 μV. Independent components (IC) were identified and rejected in an automated manner using the ADJUST tool (an EEGLAB plugin). The previously removed or missing channels were interpolated (POP_INTERP) purely for the sake of fitting the pre-processed data into the EEGLAB format for subsequent analysis and have not been used otherwise. Finally, we identified and removed the epochs containing amplitudes > 111 μV.

For further analysis a number of continuous, uncut, disjoint 10,240 samples (40 s) long epochs from each dataset were extracted.

### mMSE analysis

The rsEEG signal was analyzed using the multivariate Multiscale Sample Entropy (*mMSE*) which is an extension of the *MSE* method based on the sample entropy parameter [76] for coarse-grained (averaged) time series proposed by Costa et al. (15, 16). A full description of the *mMSE* algorithm is included in our previous work [29].

The *mMSE* analysis was conducted on the EEG activity registered from only selected electrodes. Specifically, in each participant the *mMSE* vectors were constructed based on the rsEEG signals from the channel sets corresponding to the 7 resting-state functional networks labeled after the work by Yeo et al. (2011): the default mode (DMN, T7, Fz, F4, T8, Fp2), the dorsal attention (DAN, P3, P4, C3, C4, Pz), the frontoparietal (FPN, Fp1, F4, F8, F7, F3), the limbic (LN, F7, Fp2, F8, T7, T8), the somatomotor (SMN, C3, T8, T7, C4, Cz), the ventral attention (VAN, F8, F7, Cz, T7, T8), and the visual (VN, Pz, P8, P7, O1, O2). The electrodes were assigned to each network following the probability maps provided by Giacometti et al. [38].

#### mMSE features

**Fig. 1:**
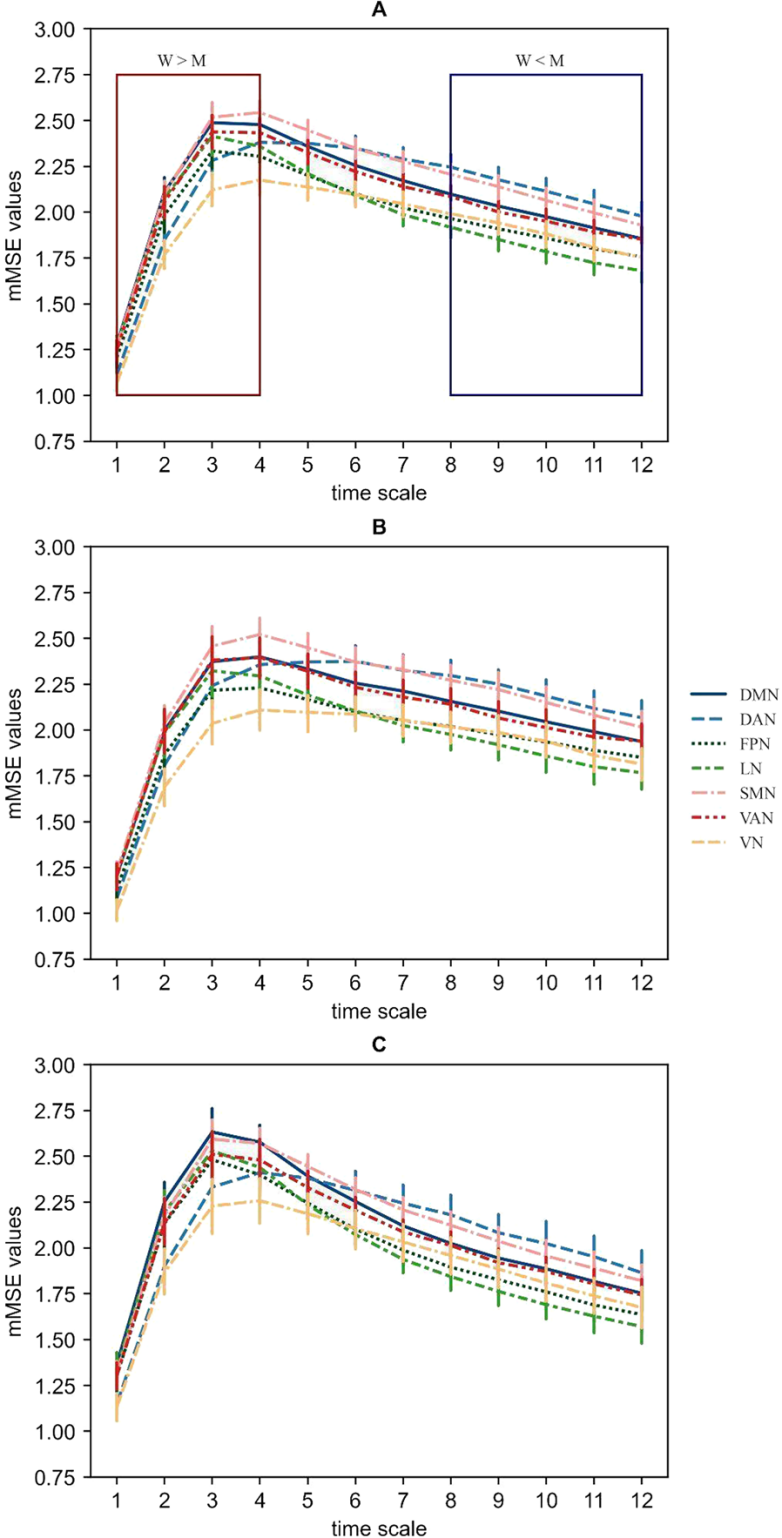
The skewed inverted-U shapes of the *mMSE* vectors for each channel set. The X-axis represents time scales (for details see Introduction Section) and the Y-axis represents the average of the *mMSE* values across the participants. Error bars represent the confidence intervals (95% CI). **A** shows the profile in the whole sample (F(1.259, 117.07) = 286.219, p < 0.001, η^2^_p_ = 0.755). The red box represents the time scales at which women showed higher resting-state EEG signal complexity than men (“W > M”) whereas the blue box includes the scales with larger complexity values in men compared to women (“W < M”). **B** shows a complexity profile for males, and **C** for females. Error bars represent the confidence intervals (95% CI). *mMSE* vectors were calculated using the following parameters for all channel sets: *m* = 2, *r* = 0.15, *p* = 4, *ε* = 12, where m is the embedding coefficient, r is the similarity threshold, p is the number of channels in a given channel set, and ε is the time scale factor. The time delay *τk* was set to 1 for *k* = 1,2,…*,p*. DMN- default mode network, DAN-dorsal attention network, FPN-frontoparietal network, LN-limbic network, SMN-somatomotor network, VAN-ventral attention network, VN-visual network

Taking into account the specific skewed inverted-U shape profile of the *mMSE* vector (Fig. 1), observed in the previous studies (e.g., [15, 29, 51, 96, 98], the following features of the *mMSE* curve were determined: the area under curve (*AUC*) to characterize the general (total) entropy level, the *MaxSlope* and the *AvgEnt* to capture the complexity at the high-frequency fine scales and at the low-frequency coarse scales respectively, as well as the *DiffEnt* to check changes of dynamics between the entropy level at the fine and coarse-grained scales, the difference in *mMSE* between the #9 and #4 timescale (*DiffEnt*) was also calculated.

The *AUC* is obtained from trapezoidal approximation of the area delimited by the *mMSE* vector and is considered as the total EEG complexity represented by the *mMSE* vector. The *MaxSlope* is the maximum pairwise difference between the first four elements (1:4 timescales) of the *mMSE* vector divided by the indices' difference. The *MaxSlope* represents the maximum complexity change of the EEG signal at the high-frequency fine-scales. The *AvgEnt* is defined as the average value of the last four elements (9:12 timescales) of the *mMSE* vector and it can be considered as representing the baseline value of entropy of the EEG signal at the low-frequency coarse-scales. These features have been already used in the analysis of neurophysiological data such as EEG or ECG (e.g., [15, 21, 96] bringing important information about the complexity of these signals. The *DiffEnt* was obtained as the difference in entropy levels between the #9 and #4 timescales for each network. The scale #4 represents the peak *mMSE* values at the fine scales whereas the scale #9 reflects the complexity level at the coarse scales where the entropy values begin to stabilize.

The scripts used to determine all these features are placed here: https://github.com/IS-UMK/complexity/tree/master/MMSE_features.

### Statistical analysis

The statistical analysis was divided into two parts. The first part contained an assessment of the quality of the data obtained using the *mMSE* algorithm, which included the evaluation of the shape of the *mMSE* curve, but also the internal consistency of the *mMSE* vectors and their features. The second part consisted of statistical tests of both channel set and s/g effects on the *mMSE* features.

In the first part (assessment of the quality of the data), both the vectors *mMSE* and their features (*AUC*, *MaxSlope, AvgEnt,* and *DiffEnt)* were analyzed. In the second part (statistical tests of hypothesis), only the *mMSE* features were used.

To evaluate the internal consistency of the *mMSE* vectors and their features, Cronbach’s alpha coefficients [18] were determined for each network, timescale (1–12), and feature (*AUC*, *MaxSlope, AvgEnt,* and *DiffEnt)*.

Next, the series of mixed ANOVA, with Greenhouse–Geisser correction, with the “Network” (7 levels) as a within-subject factor and the sex/gender as a between-subject factor was applied to check the effects of both channel set and s/g on the *mMSE* features (*AUC*, *MaxSlope, AvgEnt,* and *DiffEnt)*.

## Results

### Internal consistency of the mMSE vectors and their features

The obtained *mMSE* vectors were stable and characterized by a desired skewed inverted-U shape across the time scales (Fig. 1). The *mMSE* values stabilized at the coarse-grained time series for scale ε = 12, comparable to our previous work [29].

To evaluate the internal consistency (homogeneity) of the *mMSE* vectors and their features, the Cronbach’s alpha coefficients [18] were determined for each network, timescale (1–12) and feature (*AUC*, *MaxSlope, AvgEnt,* and *DiffEnt)*. The 5-min rsEEG data acquisition block was divided into 40-s. segments resulting in 10,240 samples (the signal was down-sampled to 256 Hz). We chose the first three uncut segments of the signal and treated each segment as an ‘item’.

**Fig. 2:**
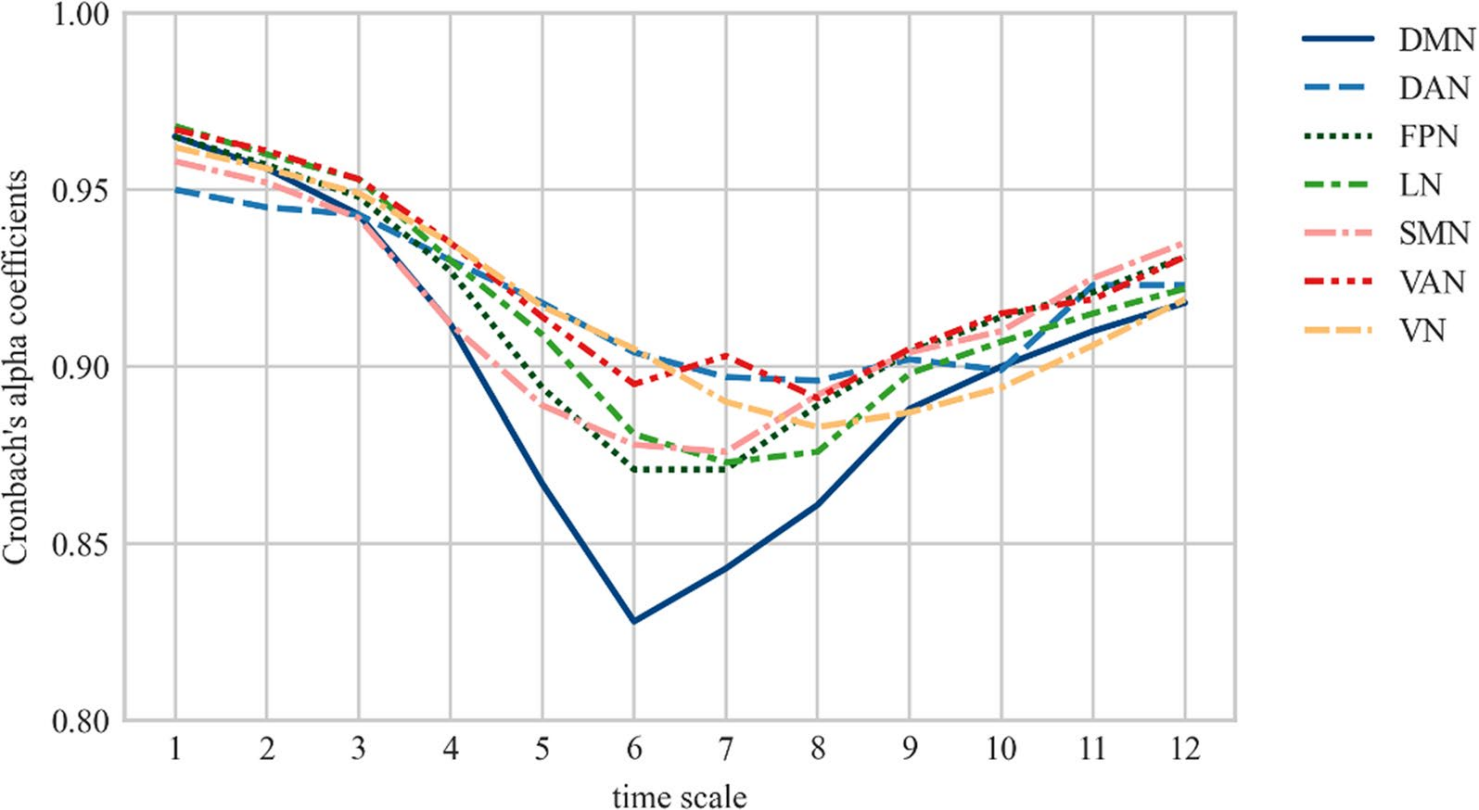
The internal consistency of each timescale for the channel sets corresponding to the resting-state networks. The X-axis represents the timescales (1–12) and the Y-axis represents the Cronbach’s alphas across the networks. DMN— default mode network, DAN-dorsal attention network, *FPN* frontoparietal network, *LN* limbic network, *SMN* somatomotor network, *VAN* ventral attention network, *VN* visual network

The Cronbach’s alpha coefficients calculated for all networks and scales (1–12) were high or very high (Cronbach’s alphas: 0.828–0.968), especially for the fine (1–4; alphas: 0.912–0.968) and coarse scales (9–12; alphas: 0.887- 0.935) (Fig. 2).

The Cronbach's alphas coefficients for particular networks and *mMSE* features are shown in Table 1. All values were in the range from 0.840 to 0.928 (for *AUC*: from 0.855 to 0.917; for *MaxSlope*: from 0.886 to 0.915; for *AvgEnt*: from 0.907 to 0.928, for *DiffEnt*: from 0.950 to 0.973), suggesting that the *mMSE* features had either a relatively high or a very high internal consistency.

**Table 1:** The internal consistency determined using the Cronbach's alpha coefficients calculated for first three segments of resting-state EEG signal, separately for each *mMSE* feature (*AUC, MaxSlope, AvgEnt, DiffEnt*) and the channel sets corresponding to seven resting-state networks.

Network	AUC	MaxSlope	AvgEnt	DiffEnt
DMN	0.855	0.896	0.912	0.971
DAN	0.917	0.840	0.923	0.950
FPN	0.890	0.903	0.923	0.973
LN	0.893	0.915	0.916	0.971
SMN	0.893	0.858	0.928	0.962
VAN	0.909	0.886	0.925	0.968
VN	0.914	0.890	0.907	0.961

*DMN* default mode network, *DAN* dorsal attention network, *FPN* frontoparietal network, *LN* limbic network, *SMN* somatomotor network, *VAN* ventral attention network, *VN* visual network

### Total rsEEG complexity and the rsEEG complexity at the fine and coarse timescales vary across the networks

The mixed ANOVA, calculated on the *AUC* values, with the “Network” (7 levels) as a within-subject factor and the “S/g” as a between-subject factor, revealed a significant main effect of the “Network” (Greenhouse–Geisser-corrected F(3.318, 308.558) = 29.484, p < 0.001, η^2^_p_ = 0.147).

The Bonferroni-corrected *post-hoc* comparisons have shown the highest *AUC* values for the SMN, DAN, and the DMN (SMN = DAN = DMN, p > 0.05) and the lowest for the VN, LN, and FPN (VN = LN = FPN, p > 0.05). In the case of SMN, the *AUC* was greater compared to the FPN, LN, VAN, VN (p < 0.001) and to the DMN (p = 0.005). The DAN was characterized by significantly (p < 0.001) higher *AUC* than the FPN, LN, and VN. The *AUC* values for the DMN and the VAN were significantly (p < 0.01) higher than for the FPN, LN, and VN. The descriptive statistics for the *AUC* features of the *mMSE* vectors are presented in Table 2 (separate for women and men, refer to Additional file 1: Appendix Table S1).

**Table 2:** Descriptive statistics for the *AUC* (area under curve), *MaxSlope*, *AvgEnt* and the *DiffEnt* features of the multivariate Multiscale Entropy (*mMSE*) vector determined for the channel sets corresponding to the seven resting-state networks (Yeo et al. 2011).

Network	M	SD	Skewness	Kurtosis
AUC				
DMN	23.448	2.790	−0.688	0.697
DAN	23.617	3.349	−0.624	0.593
FPN	21.964	3.007	−0.700	0.616
LN	21.879	3.081	−0.835	0.510
SMN	24.200	2.677	−0.941	1.752
VAN	23.066	3.033	−0.893	1.009
VN	21.381	3.554	−0.554	−0.186
MaxSlope				
DMN	0.519	0.148	0.406	−1.072
DAN	0.466	0.117	0.843	0.849
FPN	0.491	0.147	0.388	−1.097
LN	0.514	0.150	0.170	−1.132
SMN	0.517	0.131	0.567	−0.707
VAN	0.520	0.147	0.246	−1.048
VN	0.426	0.127	0.729	0.053
AvgEnt				
DMN	1.944	0.331	−0.297	−0.461
DAN	2.078	0.365	−0.368	−0.348
FPN	1.831	0.334	−0.208	−0.070
LN	1.760	0.323	−0.190	−0.298
SMN	2.032	0.324	−0.469	0.057
VAN	1.924	0.317	−0.254	−0.229
VN	1.846	0.341	−0.289	−0.522
DiffEnt				
DMN	−0.438	0.409	−0.168	−0.421
DAN	−0.195	0.303	−0.590	0.580
FPN	−0.391	0.404	−0.751	0.886
LN	−0.502	0.414	−0.312	−0.356
SMN	−0.395	0.352	−0.189	−0.093
VAN	−0.424	0.381	−0.476	0.218
VN	−0.223	0.307	−1.272	2.244

*DMN* default mode network, *DAN* dorsal attention network, *FPN* frontoparietal network, *LN* limbic network, *SMN* somatomotor network, *VAN* ventral attention network, *VN* visual network

There was a significant main effect of the “Network” for both *MaxSlope* (Greenhouse–Geisser-corrected F(4.457, 414.490) = 15.983, p < 0.001, η^2^_p_ = 0.147) and *AvgEnt* (Greenhouse–Geisser-corrected F(3.350, 311.547) = 31.949, p < 0.001, η^2^_p_ = 0.256) *mMSE* features. Table 2 contains the descriptive statistics for the *MaxSlope* and *AvgEnt* features of the *mMSE* vectors. The comparisons of the *MaxSlope* and *AvgEnt* values between particular channel sets are shown in Table 3.

**Table 3:** The differences in the *MaxSlope* and *AvgEnt* values between the channel sets corresponding to analyzed networks.

*mMSE* feature		Max Slope	Avg Ent	Max Slope	Avg Ent	Max Slope	Avg Ent	Max Slope	Avg Ent	Max Slope	Avg Ent	Max Slope	Avg Ent	Max Slope	Avg Ent
(I-J)	I	DMN		DAN		FPN		LN		SMN		VAN		VN	
J	DMN			**↓**	**↑**		**↓**		**↓**		**↑**			**↓**	
	DAN	**↑**	**↓**				**↓**	**↑**	**↓**	**↑**		**↑**	**↓**	**↓**	**↓**
	FPN		**↑**		**↑**						**↑**		**↑**	**↓**	
	LN		**↑**	**↓**	**↑**						**↑**		**↑**	**↓**	
	SMN		**↓**	**↓**			**↓**		**↓**				**↓**	**↓**	**↓**
	VAN			**↓**	**↑**		**↓**		**↓**		**↑**			**↓**	
	VN	**↑**		**↑**	**↑**	**↑**		**↑**		**↑**	**↑**	**↑**			

The *post-hoc* tests demonstrated the greatest *MaxSlope* values in the SMN, DMN, VAN, LN, and FPN (SMN = DMN = VAN = LN = FPN, p > 0.05) whereas the smallest entropy at the fine scales, significantly (p < 0.05) different from the DAN, FPN, LN, SMN, VAN, and the DMN, was found in the VN. The DAN demonstrated a significantly (p < 0.01) lower *MaxSlope* value compared to all analyzed networks except for the FPN (FPN = DAN, p > 0.05) and the VN (DAN > VN, p < 0.05)

The down-up arrows indicate the direction of significant (p < 0.05) differences between individual networks placed in columns (I) and rows (J) (mean difference, I-J). The blanks in the table indicate a lack of significant differences in the *MaxSlope* or *AvgEnt* values between particular networks. *DMN* default mode network, *DAN* dorsal attention network, *FPN* frontoparietal network, *LN* limbic network, *SMN* somatomotor network, *VAN* ventral attention network, *VN* visual network

Considering the *AvgEnt* feature, both SMN and DAN (SMN = DAN, p > 0.05) were characterized by the highest value, significantly (p < 0.001) different from other networks. The LN, FPN, and VN were characterized by the lowest entropy (LN = FPN = VN). The LN and FPN demonstrated smaller (p < 0.05) degrees of entropy at coarse scales compared to the DMN, DAN, SMN, and the VAN whereas the VN was significantly different (p < 0.001) only from the DAN and the SMN. The *AvgEnt* value for the DMN was significantly (p < 0.001) lower than for the DAN and SMN but higher compared to the FPN and LN. There were no meaningful (p > 0.05) differences between this parameter for the DMN, VAN, and the VN.

### The changes in the entropy level across the timescales

**Fig. 3:**
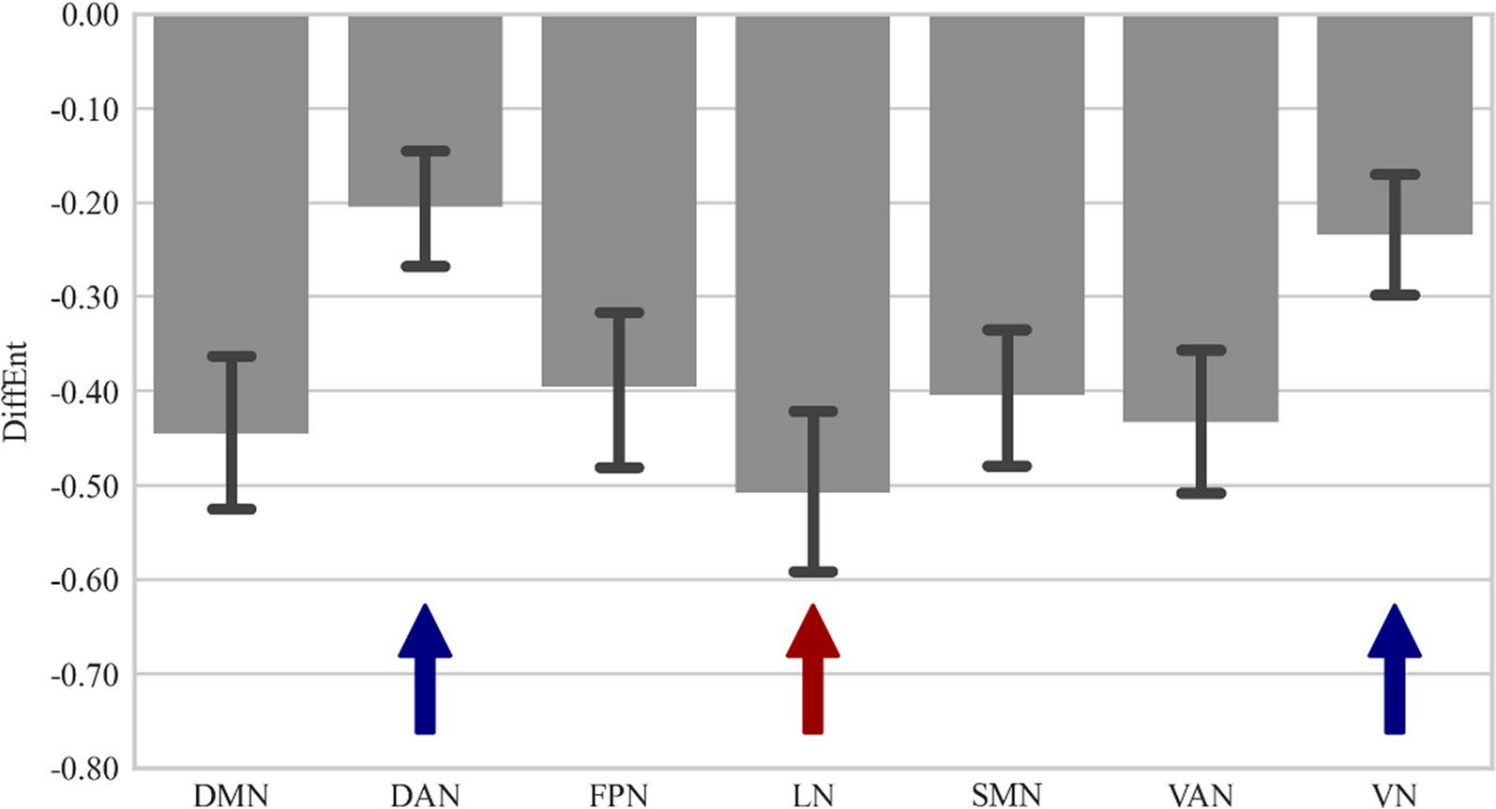
The dynamics of *MSE* changes (*DiffEnt:* the difference between #9 and #4 timescales) for particular resting-state networks across the timescales. DMN-default mode network, DAN-dorsal attention network, *FPN* frontoparietal network, *LN* limbic network, *SMN* somatomotor network, *VAN* ventral attention network, *VN*-visual network. *DiffEnt* values for the limbic network were significantly higher compared to other networks, marked on plot by the red arrow. On the other hand, both DAN and VN exhibited significantly lower values, indicated by blue arrows

The main effect of “Network” was significant (Greenhouse–Geisser-corrected F(2.762, 259.674) = 42.57, p < 0.001, η^2^_p_ = 0.312). The *post-hoc* analysis revealed the highest *DiffEnt* values for the LN whereas, the DAN and the VN (DAN = VN) demonstrated the lowest *DiffEnt values,* significantly different from all other networks. (Fig. 3; Table 2).

### Men and women are different in the rsEEG complexity at the fine and coarse timescales

For the *AUC* both the main effect of “S/g” (Greenhouse–Geisser-corrected F(1,93) = 0.159, p = 0.691, η^2^_p_ = 0.002) and the “S/g × network” interaction (Greenhouse–Geisser-corrected F(3.318, 308.558) = 0.670, p = 0.586, η^2^_p_ = 0.007) were nonsignificant.

There was a significant main effect of “S/g” for the *MaxSlope* (Greenhouse–Geisser-corrected F(1,93) = 4.485, p < 0.037, η^2^_p_ = 0.046) and the *AvgEnt* (Greenhouse–Geisser-corrected F(1,93) = 9.023, p < 0.003, η^2^_p_ = 0.088). Women showed greater *MaxSlope* values (M = 0.52 ± 0.017) than men (M = 0.472 ± 0.015) whereas men had higher *AvgEnt* (M = 1.991 ± 0.037) than women (M = 1.823 ± 0.042). For both *MaxSlope* and *AvgEnt*, the “S/g × network” interaction was nonsignificant (*MaxSlope*: F(4.457, 414.490) = 1.377, p = 0.237, η^2^_p_ = 0.015, and *AvgEnt*: (F(3.350, 311.547) = 0.304, p = 0.843, η^2^_p_ = 0.003).

We also found a significant main effect of “S/g” for the *DiffEnt* (Greenhouse–Geisser-corrected F(1,92) = 15.701, p < 0.001, η^2^_p_ = 0.146). In general, women showed greater differences between the #9 and #4 scales (M = -0.512 ± 0.049) than men (M = -0.255 ± 0.043) (see: Fig. 1 panel A and B for further exploration). The “S/g × network” interaction was non-significant (Greenhouse–Geisser-corrected F(2.775, 255.193) = 2.154, p = 0.099, η^2^_p_ = 0.023).

## Discussion

We are first to compare the rsEEG *mMSE* features between the electrode sets corresponding to the main resting-state networks. A novel result here is also an identification of the sex/gender differences in the rsEEG complexity at the fine and the coarse scales. To the best of our knowledge, this work is the second one, following Dreszer et al. [29], that quantify ed the rsEEG signal complexity using the *mMSE* algorithm, i.e. an extension of the multiscale SampEn (*MSE*) [15] to the multivariate timeseries (signals, e.g., EEG), proposed by Ahmed and Mandic (25), [3, 4]and Looney et al. [55]. Therefore, the key property of the *mMSE*, not present in the *SampEn* and *MSE* methods, is that it is designed for analysis of multivariate signals whereas the *SampEn* and *MSE* are applicable to univariate signals only. Moreover, just as the *MSE*, the algorithm, used here, examines the complexity at both fine-grained (short) and coarse-grained (long) timescales (M.U. [2–4, 55]. The shape of *mMSE* vectors in our study (Fig. 1) resembles a typical skewed inverted U-pattern [15, 16] which is believed to result from the shortest scales representing only a random signal and the longest scales reflecting a more stable system, characterized by a reduced variance [15, 62].

### The highest resting-state EEG entropy at the scalp locations corresponding to the SMN and the DAN

We found the highest, relative to other networks, *AUC* values, representing a total entropy, in the electrode sets corresponding to the SMN and the DAN (Fig. 1, Table 2). A similar location of the largest complexity (a central area) has been identified by other authors [32, 73, 95], however, the SMN also demonstrated relatively low entropy levels [47, 65, 67]. These inconsistent results may arise from different methodology, i.e. various brain signals (EEG,MEG or fMRI), resting-state conditions (eyes open or eyes closed) and algorithms used for data computation (e.g., Lempel–Ziv Complexity, *ApEn, SampEn*).

Spontaneous SMN fluctuations reflect activation of the motor system in the absence of any movement [9] whereas the DAN is involved in the top–down attention control and the expectation of objects at a particular location or with certain features [14]. The topography and strength of resting-state networks are associated with the history of network activation [22]. Therefore, the highest degrees of entropy in the SMN and DAN may reflect their increased functional connectivity with other regions or a large repertoire of possible responses to stimuli resulting from a great experience in coding features/spatial locations of the objects and motor response to these stimuli. Since the SMN and the DAN are the “task-positive” networks, their complexity levels might be more influenced by online processing and the changes in cognitive demands than the intrinsic networks such as the DMN. When there are no external tasks or stimuli, the SMN and DAN are just prepared to respond to them and in this condition there is probably no need to communicate extensively with other regions. In this context, we would be rather inclined to accept the theory postulating that greater degrees of neural complexity represent less information exchanged across brain areas [37]. Otherwise, the brain signal entropy may not directly reflect the “activity” or amount of information processing which has been already suggested by other authors (e.g., [67].

In the current study the SMN and the DAN demonstrated the greatest *AvgEnt* values corresponding to the complexity at the coarse scales (Fig. 1, Table 2) which might represent a greater capacity of these networks to process information globally, across distributed brain regions [17, 64, 94]. The long-range connections in the brain play an important role in perception [86] and information integration [48] which makes them crucial for the main functions of the SMN and the DAN. Congruently with [69] who found the highest positive correlations between the complexity at the coarse timescales and functional connectivity of the fMRI resting-state signal for the SMN and VN, the entropy at the scalp location corresponding to the SMN in our study might represent long-range interactions with other areas. The rsEEG signal from the electrode sets corresponding to the SMN also demonstrated the greatest complexity at the fine scales (Fig. 1, Table 2) suggesting the strongest, compared to other networks, short-distant functional couplings, and local synchronization across connected regions [64, 94].

Inconsistently with the previous results [59, 67, 69], the DMN in our study did not demonstrate the largest rsEEG complexity but, still, the *AUC*, *MaxSlope* and *AvgEnt* values for this network were among the highest, same as for the VAN (Fig. 1, Table 2). A relatively high entropy level in the DMN and the VAN across the timescales might be interpreted as representing greater information processing by both local and distributed neural assemblies. The DMN is considered a critical gateway for transferring information within local and across distributed networks [8, 12] which may be reflected in high degrees of entropy at the fine and the coarse scales. However, in light of the evidence showing that the DMN complexity is one of the least associated with the functional couplings [69], the interpretation of entropy in the context of functional connectivity becomes less obvious than in the case of other networks. The DMN is “active” at rest [11, 75] when its “information processing” relies on the spontaneous generation of images, voices, thoughts, and feelings that are stimulus-independent and resulted from mind wandering [58] and/or monitoring the external environment [83]. Therefore, it is even more natural to interpret a high complexity of this network in terms of extensive transitions between states and the brain’s tendency to wander [43, 63].

### The lowest resting-state EEG entropy at the scalp locations corresponding to the LN, VN and the FPN

The lowest total rsEEG entropies were observed for the channel sets linked to the LN, VN and the FPN (Fig. 1, Table 2). Similar results have been obtained by other authors in the case of VN [47, 65, 67, 99] or the LN [65, 67], however, the results suggesting the highest degrees of complexity in these two networks, compared to others, have been also reported [47, 85]. In the current study the VN showed the smallest total entropy and the complexity at both fine and coarse scales. Similarly to the VN, the LN demonstrated one of the lowest *AUC* and *AvgEnt* values. These outcomes might reflect less transitions between states [31] and/or reduced (short- and long-distant) functional connections of these networks with other regions [64]. Since the VN is mainly activated by visual stimuli, it was probably not very involved at rest when the participants are asked to keep their eyes fixated on one point in space and avoid any ocular movements. Similarly, the resting protocol favors a state of relaxation and calmness where the LN might be not particularly engaged. In this context the brain entropy would directly reflect its activity.

In contrast to other authors [59, 65, 67, 69], we demonstrated that the FPN was characterized by one of the lowest entropies (Fig. 1, Table 2). The FPN is considered as an intrinsic network, recruited by executive control tasks [28], and its complexity, similarly to the DMN, is rather expected to be among the highest at rest. A relatively low FPN entropy level, found here, may be explained referring to the method of obtaining the *mMSE* vectors. In this context a small complexity in the FPN might represent a lack of meaningful spontaneous interactions between the frontal and parietal regions forming this network. Indeed, in our previous study [29] where the *mMSE* method was applied to quantify rsEEG complexity, the parietal region was characterized by the greatest entropy whereas the frontal areas demonstrated one of the lowest entropies. Thus, we might assume that the frontal and the parietal regions, analyzed together as the FPN, will demonstrate different entropy levels than the same areas examined separately. To test this assumption, an additional *mMSE* analysis was performed on the current database but using the channel sets from our previous work [29]. We found a similar complexity pattern in case of both former and current datasets revealing the highest degrees of total complexity and the entropy at the coarse scales separately for the frontal and parietal regions (see Additional file 1: Appendix Figs. S2 and S4 ). Therefore, we might carefully conclude that the low FPN entropy in the present study results from different spatiotemporal patterns of spontaneous fluctuations in the anterior and posterior part of this network.

### Resting-state EEG entropy range between the fine and the coarse timescales

In the current study the LN showed the greatest complexity range, i.e. among the highest entropy levels at the fine scales and the lowest at the coarse scales (Fig. 1 and 3, Table 2). A similar complexity pattern was reported by McDonough and Nashiro [59] for the Cingulo-Opercular Network (CON) whose core hubs (the dorsal anterior cingulate cortex and the insula) are involved in emotional processing [27]. Such a *mMSE* profile of the LN, possibly reflecting the synchrony of neural assemblies at the fine scales and desynchrony at the coarse scales [7], may represent a process of emotional adaptation to the resting-state condition.

The DAN and the VN demonstrated relatively stable *mMSE* patterns across the timescales (had the smallest *DiffEnt* values, Fig. 3, Table 2) suggesting comparable amount and strength of local and global information processing. After reaching a relatively high complexity level both these networks showed a slow decrease of entropy (Fig. 1) which may reflect keeping balance between neural excitation and inhibition processes.

The DAN could be also considered as a separable, stable, internally more coherent, module in the brain [42]. The complexity of the task-positive networks such as the DAN and the VN, may change more in a task than at rest [59]. Therefore, a small differences in the entropy level at the fine and the coarse scales (low *DiffEnt* values) in the DAN and the VN may result from a relatively stable amount of visual stimulation and recruited cognitive resources during the resting state.

Interestingly, the DMN and the VAN in our study had almost overlapping *mMSE* vector shapes (Fig. 1) showing among the highest *MaxSlope* and *AvgEnt* values relative to other networks (Table 2). This effect is hard to explain in terms of sharing the same channels by these networks (the DMN and the VAN had only two common positions: T7 and T8). It is of note that we analyzed the rsEEG signal from the electrode set corresponding to the VAN, extracted by Yeo et al. (2011), and this network includes both the CON and the “classic” VAN which have demonstrated strong synchronization at rest [27, 71] possibly reflected in the great degrees of rsEEG entropy.

The DMN is considered as a part of the task-negative system whereas the VAN/CON is identified as a task-positive network [52]. In this context the DMN and the VAN/CON were supposed to indicate some inverse (anti-correlated) functionality resulting from the allocation of limited resources. As a support for this claim McDonough and Nashiro [59] found the lowest complexity at the fine scales and the highest entropy at the coarse scales in the DMN whereas for the CON the inverse neural complexity profile was obtained. In our study similar *mMSE* patterns in the DMN and the VAN may result from the function that they both share, e.g. tonic alertness [11, 78] which is highly involved during the resting state.

In our study the SMN and the LN had similar *mMSE* vector shape to the DMN/VAN (Fig. 1). While the VAN and the LN signals were acquired from very similar sets of electrodes (which may, to some extent, explain the similarity of their rsEEG complexity profiles), for the DMN or the SMN this was not the case. We might speculate then that the resemblance of *mMSE* patterns between particular channel sets (networks) might reflect their strong interactions or interdependencies. Conversely, different entropy patterns in given networks might represent weak connections between these systems. Therefore, in the present study, similar complexity profiles, especially in the DMN and the VAN and, to a lesser extent, in the SMN and the LN, might suggest the increased communication between these networks at rest.

### Higher resting-state EEG complexity at the fine timescales and lower at the coarse scales in women compared to men

Although we did not find any significant s/g differences in the *AUC* values, corresponding to the total rsEEG complexity, women, relative to men, showed higher *MaxSlope* values (the fine scales) but lower *AvgEnt* values (the coarse scales) (Fig. 1). Since we are not aware of any work presenting the s/g differences in the *mMSE* values, we decided to quantify the complexity of the current data using exactly the same method and regions of interest as in our previous work [29]. The detailed results of this re-analysis were shown in the Additional file 1: Appendix Figs. S2–4. Basically, we found a reproducible pattern of the s/g differences in the entropy level at the fine and the coarse timescales: for both datasets, women, compared to men, produced significantly higher *MaxSlope* values in the frontal areas and men showed greater *AvgEnt* values than women for all analyzed channel sets. Considering the *AUC*, the outcomes across both our studies were inconsistent: females demonstrated a greater total entropy level than males only for our previous dataset and we did not observe such s/g differences in the current study.

The way how the *MaxSlope* is calculated (the maximum change in the EEG signal complexity at the fine scales) allows to compare this parameter with the entropy, determined using other methods and measured at a single timescale. In this context, the s/g differences in the *MaxSlope* value are congruent with some previous findings [32, 50, 72, 98]. Higher degrees of entropy at the short scales and lower complexity at the long scales in women might reflect enhanced information processing in local neural assemblies but reduced large-scale interactions in the resting brain [64, 93]. The previous resting-state fMRI evidence suggesting a greater overall brain integration of specialized information at a global network level in males and higher segregation, i.e. specialized processing of the brain at a local level, in females supports the aforementioned interpretation of the brain complexity [5, 45, 79, 89]. Some authors, however, reported the opposite s/g differences in the short- and long-range functional connectivity [33, 100]. There is also evidence on greater anatomical connectivity and higher both local and global efficiency of the cortical networks in women compared to men [39].

Greater neural complexity at the coarse scales as reflecting more distributed organization of the brain in men might result from optimal functioning of specialized and complex processes such as visuospatial imagery or orientation, which recruit long-distance connections. For example, the mental rotation tasks, where men often outperformed women [39, 41, 44], require visualization of the rotation of objects in space and, then, correctly matching them with exemplars which involve testing and comparisons before a decision is made. Furthermore, previous studies have revealed that men do not exhibit a higher local efficiency in visuospatial processing regions [39] suggesting that men’s superior performance in such tasks may also rely on the long-range interactions of these areas. The greater local information processing in females, on the other hand, might optimize functions that require synchronization across local networks such as those supporting verbal fluency in which women achieve better scores [36, 80, 81]. The s/g differences in the neural complexity at the fine and the coarse scales (and corresponding local and global network processing) might also reflect a predisposition of one of the sexes to develop certain disorders including autism which occur the 5–10 times more often in men than in women and is characterized by reduced local functional connections in the brain [91].

In the present study we did not find any significant interaction effects between the s/g factor and the channel sets corresponding to the resting-state networks, distinguished by Yeo et al. (2011). The s/g differences in the neural complexity at the short and the long scales, were observed in the whole brain, not in particular networks. However, when we analyzed the current data using the same regions of interest as in our previous study [29], it turned out that women, relative to men, showed higher *MaxSlope* values in the frontal areas which is, basically, in line with other findings [1, 72]. In the current study a lack of specific scalp locations with significant s/g differences in the rsEEG entropy level might result from overlapping channel sets forming particular networks. For example, the frontal electrodes from our previous work [29], where women showed higher *MaxSlope* values than men, are here distributed among 4 networks (FPN, DMN, LN and VAN). Therefore, it is possible that the signals from these channels contributed significantly to the temporal dynamics of all aforementioned networks producing the cumulative effect observed at the whole brain level.

**Fig. 4:**
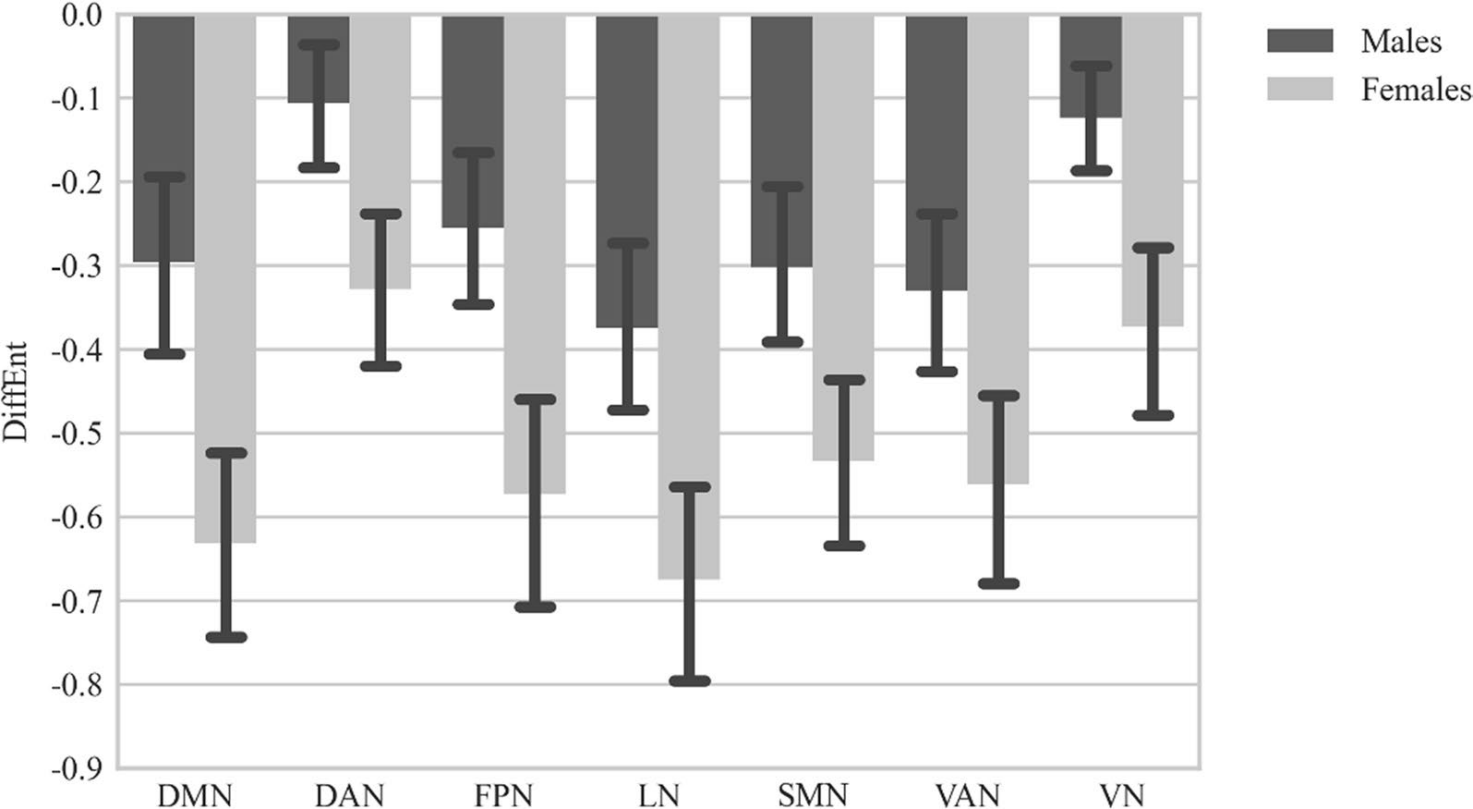
The s/g-related differences in the dynamics of MSE changes (*DiffEnt*: the difference between #9 and #4 timescales) for particular resting-state networks across the timescales. *DMN* default mode network, *DAN* dorsal attention network, *FPN* frontoparietal network, *LN* limbic network, *SMN* somatomotor network, *VAN* ventral attention network, *VN* visual network

Interestingly, in our study women showed higher *DiffEnt* values (less stable *mMSE* profiles) than men in all analyzed networks (Fig. 4). These outcomes may suggest comparable levels of local and global information processing in male brain during the resting state and the advantage of short-distance over the long-distance interactions in females.

### Limitations of the study and further directions

The present outcomes should be interpreted in light of several limitations. The most serious concern the validity and reliability of the analyzed variables. The particular aspects of current study burdened by the most important limitations are listed below.

#### Interpretation of entropy values at the fine and the coarse scales

Referring the brain signal complexity at the short and the long scales to local and global information processing respectively, has already met some criticism [49, 69] and needs further verification. In future studies it would be beneficial to take into account the anatomical data while interpreting the neural complexity results, especially information about the white matter microstructure since the white matter integrity has been already related to the network complexity at both fine and coarse scales [60]. Furthermore, the coarse-graining procedure acts as a moving average low-pass filter that impedes separation of particular frequency bands from the signal. Therefore, the relationship between the *mMSE* and the frequency content of EEG signal is difficult to determine [17], although a certain progress has been made recently in this area [49].

#### Reliability of the *mMSE* vectors and their features

In our study stability of the *mMSE* vectors has been checked using an internal consistency method. The obtained results suggest either a high or a very high reliability, both in the case of timescales and features of the *mMSE* vectors. To date, attempts to check the internal consistency of complexity indexes have been made in a few EEG studies (e.g., [89]. However, it does not exhaust the need for in-depth research on the reliability of *MSE* measures. In future research the test–retest stability should be determined (comparable to some fMRI studies, e.g., [69]).

#### The necessity to replicate the study results

The present study is the second in the literature (after our previous work) where the *mMSE* algorithm was used to describe the dynamics of resting-state EEG activity at the short and the long timescales and is also the first attempt to use the multivariate method to describe the dynamics of the brain networks during the resting state. Hence, it would be strongly recommended to ensure to what extent obtained results could be replicated on a different sample. Being aware of this problem in this work we re-analyzed the current results with the use of electrode sets from our previous work [29] and demonstrated a substantial consistency in both our studies (Additional file). In future research, however, it would be desired to replicate the results also from the brain network perspective.

#### Weaknesses of the methods: the *mMSE* algorithm and the proximity maps

##### Limitations of the *mMSE* algorithm

In the present study the same *mMSE* algorithm with the same parameters (EEG signal segmented into multivariate time series of length n = 1024, with *m* = 2, *r* = 0.15, *p* = 4, ε = 12, τk was set to 1 for *k* = 1,2,…,*p,* where *m* is the embedding coefficient, *r* is the similarity threshold, *p* is the number of channels in a given channel set, and *ε* is the timescale factor, *τk* is the time delay) as in our previous work [29] was used which allows for direct result comparisons. On the other hand, both these analyses share the same limitations of the *mMSE* algorithm which was already discussed elsewhere [29]. (Sec. 4.3)

##### A lack of direct localization of EEG sources/problem of proximity maps

An important limitation of our analysis is the selection of channel sets based on the average electrode proximity maps provided by Giacometti et al. [38] without a direct localization of EEG sources in our dataset. Unfortunately in the present study the MRI anatomical data useful for proper source localization were not available. A lack of EEG source locations did not allow to exact identification of the networks, therefore, the proximity parcellations can ONLY be treated as an approximation.

Since our sample comprised only young healthy adults, the models (the regions of interest from the standard brain atlases), used by Giacometti et al. [38], might be a quite good approximation. However, in the case of more specific groups, these models could be hardly applied (a decreased effect size, [25]. In our opinion this method is only applicable when we may assume that the variability of anatomical locations of EEG sources will be comparable across participants, i.e. mainly in the case of a relatively homogenous sample, e.g. healthy young adults. In future research on the resting networks complexity, tested using EEG, it would be reasonable to perform the source localization.

##### Electrode sets

We should also mention about the electrode sets used in our study. They correspond to 7 main resting networks determined using the algorithm developed by Giacometti et al. [38] and have the important disadvantage of including overlapping channels forming particular networks. It would be advisable to give up the overlapping electrodes and assign each channel to only one network. Otherwise, it would be recommended to resign from including to the analysis both LN and VAN channel sets differing in only one electrode. However, this change would cause a serious interference in the original parcellation which was created based on the localization and reconstruction of the EEG sources. In this case the confirmation of the procedure validity would be required.

Finally, our study was not free from limitations that also exist in almost every resting-state EEG study on the s/g differences (e.g., lack of controlling for the menstrual cycle [61].

## Conclusions

We found the highest overall resting-state EEG complexity, measured using the *mMSE* method, at the channel sets corresponding to both intrinsic (DMN) and extrinsic (SMN and DAN) networks, which is basically in line with previous studies. Surprisingly, in contrast to other findings, the FPN was characterized by one of the lowest total entropy which may be explained by specificity of the *mMSE* algorithm. A novel result of this study is an indication of the resting-state EEG dynamics across different electrode sets (networks) and the timescales. Furthermore, our study highlights the importance of the neural complexity range while interpreting the outcomes. For the first time we have shown the sex/gender differences in the spontaneous EEG signal complexity at the fine and the coarse scales. Women, relative to men, demonstrated a higher degree of entropy at the short scales and lower at the long scales which might be interpreted in terms of an increased local and decreased global information processing in the female compared to the male brain.

### Supplementary Information

**Additional file 1: Figure A1**. Age-related differences between females and males. **Figure A2 **Overall complexity level (*AUC*) measured by mMSE for nine areas of the scalp. Channel set effect: F(8,87) =139.93, p <0.001, η²_p_ = 0.928.** Figure A3 **Fine-scale complexity (*MaxSlope*) values for nine areas of the scalp. Channel set effect: F(8,87)  =55.326, p < 0.001, η²_p_ = 0.836.** Figure A4 **Coarse-grained time scales complexity (*AvgEnt*) for nine areas of the scalp. Channel set effect: F(8,87) = 125.049, p < 0.001, η²_p_ = 0.920.** Figure A5** *mMSE *features (a. *AUC*, b. *MaxSlope*, c. *AvgEnt*) for particular resting-state networks across the timescales and three segments (bars marked with different textures: segment #1 (stripes), segment #2 (checkered), segment #3 (dotted)). DMN-default mode network, DAN-dorsal attention network, FPN-frontoparietal network, LN-limbic network, SMN-somatomotor network, VAN-ventral attention network, and VN-visual network.** Figure A6** The dynamics of *MSE *changes (*DiffEnt:* the difference between #9 and #4 timescales) for particular resting-state networks across the timescales and three segments (bars marked with different textures: segment #1 (stripes), segment #2 (checkered), segment #3 (dotted)). DMN-default mode network, DAN-dorsal attention network, FPN-frontoparietal network, LN-limbic network, SMN-somatomotor network, VAN-ventral attention network, and VN-visual network. *DiffEnt* values for the limbic network were significantly higher compared to other networks. On the other hand, both DAN and VN exhibited significantly lower values.** Figure A7** The s/g-related differences in the dynamics of MSE changes (*DiffEnt*: the difference between #9 and #4 timescales) for particular resting-state networks across the timescales. DMN-default mode network, DAN-dorsal attention network, FPN-frontoparietal network, LN-limbic network, SMN-somatomotor network, VAN-ventral attention network, VN-visual network. Males - bars marked with beige; females - bars marked with checkered texture.** Table A1** Descriptive statistics for the *AUC* (area under curve), *MaxSlope*, *AvgEnt *and the *DiffEnt *features of the multivariate Multiscale Entropy (*mMSE*) vector determined for the channel sets corresponding to the seven resting-state networks (Yeo et al., 2011) for females (**A**) and males (**B**), separately. *DMN* default mode network, *DAN* dorsal attention network, *FPN* frontoparietal network, *LN* limbic network, *SMN* somatomotor network, *VAN* ventral attention network, *VN* visual network.

## Data Availability

Data/code availability statement: EEG dataset and database of *mMSE* vectors and features http://www.is.umk.pl/~tpiotrowski/EEG_complexity_neuroimage/. Preprocessing script: https://github.com/IS-UMK/complexity/tree/master/Preprocessing. Scripts calculating mMSE vectors: https://github.com/IS-UMK/complexity/tree/master/MMSE_vectors. Scripts calculating mMSE features: https://github.com/IS-UMK/complexity/tree/master/MMSE_features. Code will be shared with no restrictions upon request.

## Abbreviations

ApEn: The approximate entropy
SampEn: The sample entropy
MSE: Multiscale Entropy
mMSE: Multivariate Multiscale Entropy
rsEEG: The resting-state EEG
s/g: The sex/gender
DMN: Default mode network
DAN: Dorsal attention network
FPN: Frontoparietal network
LN: Limbic network
SMN: Somatomotor network
VAN: Ventral attention network
VN: Visual network
AUC: The area under curve, the general (total) EEG entropy level
MaxSlope: Th complexity of EEG signal at the high-frequency fine scales
AvgEnt: The complexity of EEG signal at the the low-frequency coarse scales
DiffEnt: Stability of the complexity EEG signal across timescales
