# Supplementary material for: Multivariate multiscale entropy (mMSE) as a tool for understanding the resting-state EEG signal dynamics: the spatial distribution and sex/gender-related differences
Source: Behav Brain Funct. 2023 Oct 5;19:18. doi: 10.1186/s12993-023-00218-7 (PMC10552392; doi:10.1186/s12993-023-00218-7)
Supplement: Supplementary file 1 — Additional file 1: Figure A1. Age-related differences between females and males. Figure A2 Overall complexity level (AUC) measured by mMSE for nine areas of the scalp. Channel set effect: F(8,87) =139.93, p <0.001, η²p = 0.928. Figure A3 Fine-scale complexity (MaxSlope) values for nine areas of the scalp. Channel set effect: F(8,87) =55.326, p < 0.001, η²p = 0.836. Figure A4 Coarse-grained time scales complexity (AvgEnt) for nine areas of the scalp. Channel set effect: F(8,87) = 125.049, p < 0.001, η²p = 0.920. Figure A5 mMSE features (a. AUC, b. MaxSlope, c. AvgEnt) for particular resting-state networks across the timescales and three segments (bars marked with different textures: segment #1 (stripes), segment #2 (checkered), segment #3 (dotted)). DMN-default mode network, DAN-dorsal attention network, FPN-frontoparietal network, LN-limbic network, SMN-somatomotor network, VAN-ventral attention network, and VN-visual network. Figure A6 The dynamics of MSE changes (DiffEnt: the difference between #9 and #4 timescales) for particular resting-state networks across the timescales and three segments (bars marked with different textures: segment #1 (stripes), segment #2 (checkered), segment #3 (dotted)). DMN-default mode network, DAN-dorsal attention network, FPN-frontoparietal network, LN-limbic network, SMN-somatomotor network, VAN-ventral attention network, and VN-visual network. DiffEnt values for the limbic network were significantly higher compared to other networks. On the other hand, both DAN and VN exhibited significantly lower values. Figure A7 The s/g-related differences in the dynamics of MSE changes (DiffEnt: the difference between #9 and #4 timescales) for particular resting-state networks across the timescales. DMN-default mode network, DAN-dorsal attention network, FPN-frontoparietal network, LN-limbic network, SMN-somatomotor network, VAN-ventral attention network, VN-visual network. Males - bars marked with beige; females - bars marked with checkered te [file 12993_2023_218_MOESM1_ESM.pdf]

Appendix for:

Multivariate Multiscale Entropy (*mMSE*) as a tool for understanding the resting-state EEG signal dynamics: the spatial distribution and sex/gender-related differences

Monika Lewandowska[1], Krzysztof Tołpa[1], Jacek Rogala[2], Tomasz Piotrowski[3], Joanna Dreszer[1*]

[1] Institute of Psychology, Faculty of Philosophy and Social Sciences, Nicolaus Copernicus University in Torun, Gagarina 39 Street, 87-100 Torun, Poland
[2] Faculty of Physics, University of Warsaw, Pasteur 5 Street, 02-093 Warsaw, Poland
[3] Institute of Engineering and Technology, Faculty of Physics, Astronomy and Informatics, Nicolaus Copernicus University in Torun, Grudziądzka 5 Street, 87-100 Torun, Poland.

*Corresponding author
E-mail: jdreszer@umk.pl (JD)
Department of Clinical Psychology and Neuropsychology
Institute of Psychology
Faculty of Philosophy and Social Sciences
Nicolaus Copernicus University in Torun
Gagarina 39 Street, 87-100 Torun, Poland

A.1. Age-related differences between women (M = 25.31, SD = 4.91) and men (M = 26; SD = 4.26) were statistically nonsignificant, U Mann- Whitney test: U = 900.5, p = .109 (see Fig.A1.).

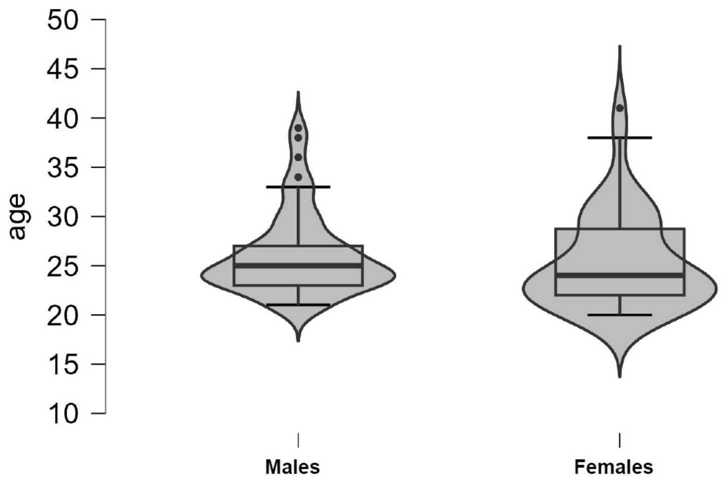

Fig. A1. Age-related differences between females and males.

Table A1. Descriptive statistics for the *AUC* (area under curve), *MaxSlope*, *AvgEnt* and the *DiffEnt* features of the multivariate Multiscale Entropy (*mMSE*) vector determined for the channel sets corresponding to the seven resting-state networks (Yeo et al., 2011) for females (A.) and males (B.), separately. DMN-default mode network, DAN-dorsal attention network, FPN-frontoparietal network, LN-limbic network, SMN-somatomotor network, VAN-ventral attention network, VN-visual network.

A. Females

**Sex/Gender**	**FEMALES**			
**Network**	**M**	**SD**	**Skewness**	**Kurtosis**
	**AUC**			
**DMN**	23.460	2.530	-0.441	0.501
**DAN**	23.330	3.412	-0.749	1.853
**FPN**	21.988	2.740	-0.920	0.392
**LN**	21.796	2.527	-0.548	0.689
**SMN**	23.890	2.172	-0.606	-0.154
**VAN**	22.880	2.477	-0.579	0.110
**VN**	21.474	3.481	-1.086	0.961
**MaxSlope**				
**DMN**	0.560	0.150	0.038	-1.043
**DAN**	0.481	0.120	0.403	0.158
**FPN**	0.534	0.159	-0.035	-1.311
**LN**	0.541	0.143	0.030	-1.226
**SMN**	0.547	0.132	0.340	-1.291
**VAN**	0.532	0.140	0.116	-1.416
**VN**	0.447	0.133	0.524	-0.225
**AvgEnt**				
**DMN**	1.850	0.317	-0.197	-0.837
**DAN**	1.981	0.374	-0.160	-0.048
**FPN**	1.728	0.304	-0.486	-0.313
**LN**	1.663	0.278	-0.556	-0.358
**SMN**	1.926	0.286	-0.587	-0.645
**VAN**	1.834	0.266	-0.578	-0.229
**VN**	1.777	0.335	-0.547	-0.546
**DiffEnt**				
**DMN**	-0.632	0.369	-0.010	-0.094
**DAN**	-0.328	0.317	-0.833	0.064
**FPN**	-0.573	0.416	-0.762	0.890
**LN**	-0.676	0.379	-0.368	-0.053
**SMN**	-0.534	0.338	-0.431	-0.465
**VAN**	-0.561	0.382	-0.608	0.175
**VN**	-0.373	0.343	-1.087	1.046

B. Males

Sex/Gender	MALES			
**Network**	**M**	**SD**	**Skewness**	**Kurtosis**
	**AUC**			
**DMN**	23.437	3.005	-0.805	0.749
**DAN**	23.904	3.308	-0.535	-0.387
**FPN**	21.939	3.228	-0.595	0.700
**LN**	21.963	3.481	-0.933	0.265
**SMN**	24.510	3.001	-1.177	2.251
**VAN**	23.252	3.425	-1.042	1.073
**VN**	21.288	3.642	-0.200	-0.766
		**MaxSlope**		
**DMN**	0.487	0.140	0.739	-0.732
**DAN**	0.454	0.113	1.273	2.145
**FPN**	0.457	0.128	0.677	-0.634
**LN**	0.493	0.154	0.335	-1.004
**SMN**	0.494	0.127	0.808	0.071
**VAN**	0.511	0.154	0.363	-0.814
**VN**	0.408	0.121	0.925	0.602
		**AvgEnt**		
**DMN**	2.019	0.325	-0.461	0.033
**DAN**	2.155	0.341	-0.517	-0.435
**FPN**	1.912	0.336	-0.226	-0.032
**LN**	1.836	0.338	-0.289	-0.401
**SMN**	2.117	0.330	-0.709	0.784
**VAN**	1.995	0.339	-0.413	-0.250
**VN**	1.900	0.339	-0.141	-0.768
		**DiffEnt**		
**DMN**	-0.296	0.388	-0.429	-0.203
**DAN**	-0.106	0.277	-0.421	0.595
**FPN**	-0.255	0.335	-0.436	-0.365
**LN**	-0.375	0.399	-0.441	-0.562
**SMN**	-0.301	0.349	-0.249	0.134
**VAN**	-0.330	0.364	-0.397	-0.125
**VN**	-0.123	0.242	-1.143	2.192

Supplementary analysis A2: re-analysis of s/g differences in the *mMSE* values for the channel sets corresponding to the scalp areas

We re-analyzed the current data using nine channel sets from our previous study (Dreszer et al., 2020).

Channels were located in the frontal (*F*: F7, F8, F3, F4), frontal left (*FL*: FP1, F7, F3, FC3), frontal right (*FR*: FP2, F8, F4, FC4), central (midline) (*C*: Fz, Cz, Pz, Oz), parietal (*P*: P3, P4, P7, P8), parietal left (*PL*: P7, P3, O1, PO3), and parietal right (*PR*: P8, P4, O2, PO4), middle left (*ML*: T7, C3, Cp5, Cp1), middle right (*MR*: T8, C4, Cp6, Cp2), regions of the scalp.

mMSE feature analysis (a mixed MANOVA) showed the main effects of channel set (region) for *AUC* (channel set: $F(8,87) = 139.93$, $p < 0.001$, $\eta^2_p = 0.928$), for *MaxSlope* and *AvgEnt* both the main effects of s/g and channel set (region); for *MaxSlope* (s/g: $F(1,94) = 6.680$, $p < 0.01$, $\eta^2_p = 0.066$, F > M; channel set: $F(8,87) = 55.326$, $p < 0.001$, $\eta^2_p = 0.836$), and *AvgEnt* (s/g: $F(1,94) = 9.428$, $p < 0.01$, $\eta^2_p = 0.091$, F < M; channel set: $F(8,87) = 125.049$, $p < 0.001$, $\eta^2_p = 0.920$). For *AUC* effect of s/g wasn't significant: $F(1,94) = 0.290$, $p = 0.592$, $\eta^2_p = 0.003$.

The interaction effects for both *AUC* $F(8,87) = 2.088$, $p = 0.045$, $\eta^2_p = 0.161$), and *MaxSlope* (s/g x channel set: $F(8,87) = 1.936$, $p = 0.064$, $\eta^2_p = 0.151$, tendency level) were significant. The interaction effect for *AvgEnt* has not reached the significance level (s/g x channel set: $F(8,87) = 1.649$, $p = 0.123$, $\eta^2_p = 0.132$).

Effect of channel sets

For *AUC*, the highest values of complexity were obtained for the following sets: F, C, P, MR, ML, and the lowest for FL, FR, PL, PR. For *MaxSlope*, the highest for F and ML, and MR; the lowest for PL and PR were revealed. For *AvgEnt*, the highest values of complexity were obtained for sets F, C, P, MR, ML, and the lowest for FL, FR, PL, PR.

Effect of s/g

Generally, in women, there was greater brain signal complexity at fine-grained scales (*MaxSlope)* than in men ($p < 0.01$). In men, there was greater entropy at coarse-grained timescales (*AvgEnt*) ($p < 0.01$).

Interaction effects of channel set x s/g

Taking into account the interaction effect (s/g x channel set: $F(8,87)= 1.936$, $p = 0.064$, $\eta^2_p= 0.151$, tendency level), it should be emphasized that the s/g differences were observed at fine-scales (*MaxSlope)* for the following regions: F, FL, FR, on tendency level. In women, there was greater brain signal complexity at fine-grained scales for F, FL, and FR regions than in males. At coarse-grained timescales (*AvgEnt*) interaction effect (s/g x channel set: $F(8,87)= 1.649$, $p = 0.123$, $\eta^2_p = 0.132$) wasn't significant. For *AUC*, the interaction effect was significant $F(8,87)= 2.088$, $p = 0.045$, $\eta^2_p = 0.161$), but only PL (the lower level of complexity) differed significantly from other regions.

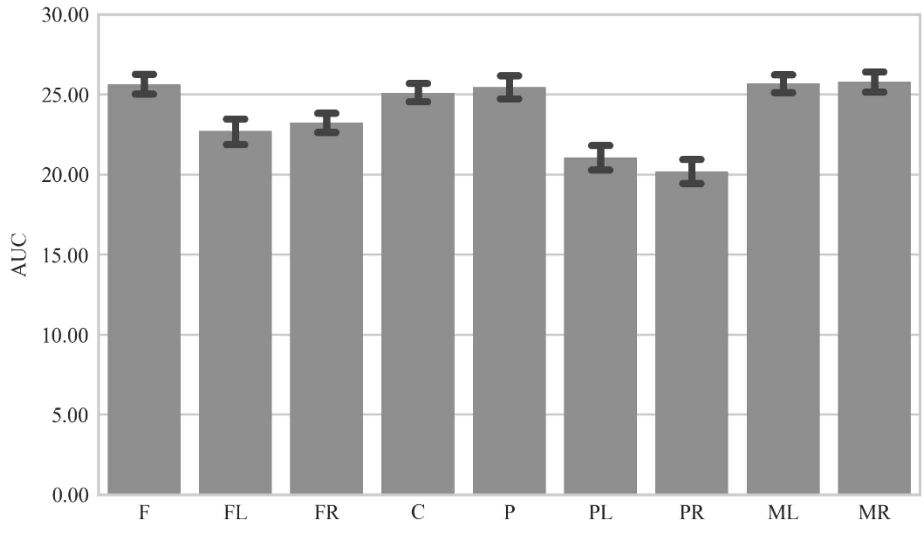

Fig. A.2. Overall complexity level (*AUC*) measured by mMSE for nine areas of the scalp. Channel set effect: $F(8,87) =139.93$, $p <0.001$, $\eta^2_p = 0.928$.

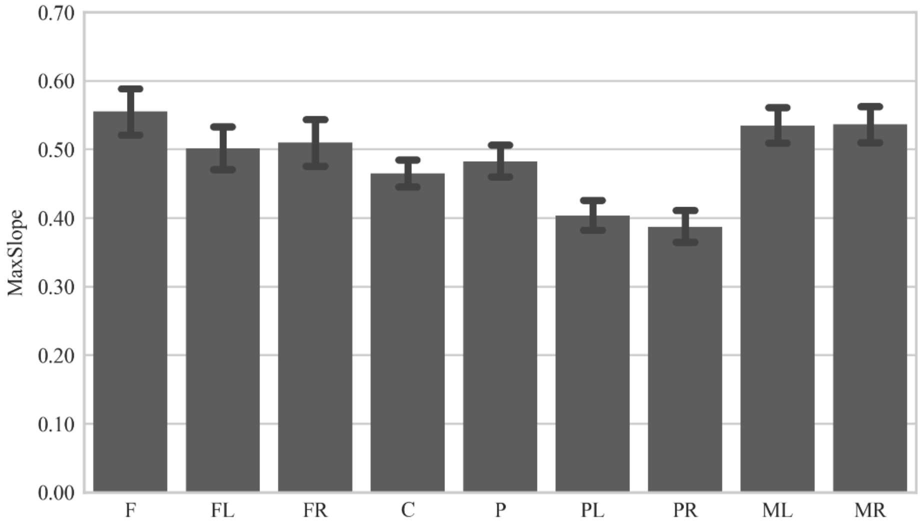

Fig. A.3. Fine-scale complexity (*MaxSlope*) values for nine areas of the scalp. Channel set effect: $F(8,87) = 55.326$, $p < 0.001$, $\eta^2_p = 0.836$.

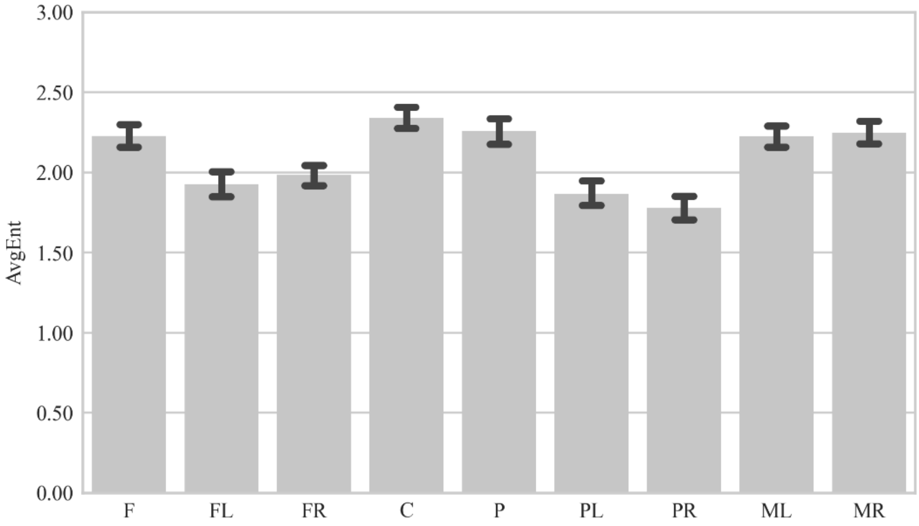

Fig. A.4. Coarse-grained time scales complexity (*AvgEnt*) for nine areas of the scalp. Channel set effect: $F(8,87) = 125.049$, $p < 0.001$, $\eta^2_p = 0.920$.

To sum up, there was the greatest general brain signal complexity (*AUC*) for the P set, and the lowest complexity was observed for FL, FR, Pl, and PR sets. No significant differences in *AUC* were revealed for F, C, ML, and MR sets. For *MaxSlope* the greatest level of complexity were observed for F, FL, P, and ML, MR channel sets. For *AvgEnt* the greatest level of complexity was observed for C and P sets, the lowest for FL, FR, PL, PR.

For *AUC*, the highest values of complexity were obtained for the following sets: F, C, P, MR, ML, and the lowest for FL, FR, PL, PR. For *MaxSlope*, the highest for F and ML, and MR; the lowest for PL and PR were revealed. For *AvgEnt*, the highest values of complexity were obtained for sets F, C, P, MR, ML, and the lowest for FL, FR, PL, PR.

Both the previous (Dreszer et al., 2020) and the current study showed s/g differences in *MaxSlope* (F > M) and *AvgEnt* (F < M, in the previous study - on tendency level). Differences between men and women for *AUC* were shown only in the previous study (Dreszer et al., 2020), this effect was not confirmed in the re-analysis taking into account the nine sets of electrodes in the current study.

The differences between men and women at fine scales were statistically significant mainly for the channels from the frontal sets: F, FL, FR (the effect was demonstrated in both studies). The previous study (Dreszer et al., 2020) also showed a difference in the MR and P sets, which were not shown in the current study.

References:

Dreszer, J., Grochowski, M., Lewandowska, M., Nikadon, J., Gorgol, J., Bałaj, B., Finc, K., Duch, W., Kałamała, P., Chuderski, A., Piotrowski, T., 2020. Spatiotemporal complexity patterns of resting-state bioelectrical activity explain fluid intelligence: Sex matters. Hum. Brain Mapp. 41, 4846–4865. https://doi.org/10.1002/hbm.25162

Supplementary analysis A3.

We repeated the analysis taking into account three segments of *mMSE* features.

We replicated the results for *AUC*, *MaxSlope*, *AvgENT*, and *DiffENT*.

The choice of the number of segments was strongly dependent on the number of participants in whom these segments were identified. The 5-min rsEEG data acquisition block was divided into 40-sec. segments resulting in 10240 samples (the signal was down-sampled to 256 Hz). We chose the first three uncut segments of the signal and treated "segment" as a within-subject factor in mixed ANOVA.

A3.1. Total rsEEG complexity and the rsEEG complexity at the fine and coarse timescales vary across the networks

The mixed ANOVA, calculated on the *AUC* values, with the "Network" (7 levels) and "Segment" (3 levels) as a within-subject factor and the "S/g" as a between-subject factor, revealed a significant main effect of the "Network" (Greenhouse-Geisser-corrected $F(3.36, 309.467)=36.002$, $p<0.001$, $\eta^2_p=0.281$) and significant interaction effect "Network x segment" (Greenhouse-Geisser-corrected $F(4.32, 397.795)=3.456$, $p<0.01$, $\eta^2_p=0.04$) (Fig. A.5., a.).

There was a significant main effect of the "Network" and "Segment" for *MaxSlope* (Greenhouse-Geisser-corrected for "Network": $F(3.628, 322.927)=31,842$, $p<0.001$, $\eta^2_p=0.264$; "Segment": $F(1.66, 148.084)=4.55$, $p=0.017$, $\eta^2_p=0.049$) and "Network" for *AvgEnt* (Greenhouse-Geisser-corrected "Network": $F(3.35, 308.567)=34.882$, $p<0.001$, $\eta^2_p=0.275$) *mMSE* features (Fig. A.5., b. and c.). Moreover, the interaction effect "Network x Segment" was significant for *AvgEnt* (Greenhouse-Geisser-corrected: $F(4.883, 449.246)=5.386$, $p<0.001$, $\eta^2_p=0.055$).

a. AUC

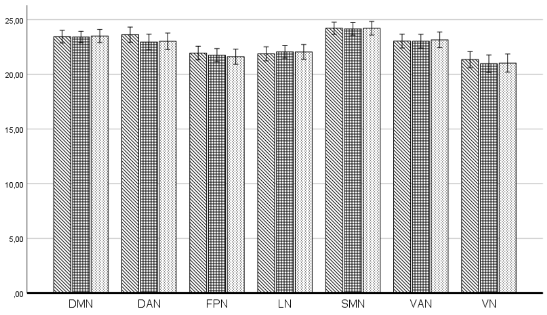

b. MaxSlope

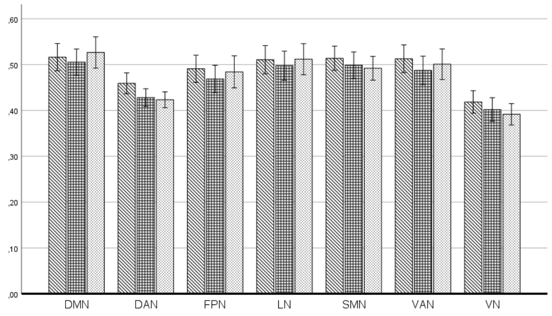

c. AvgEnt

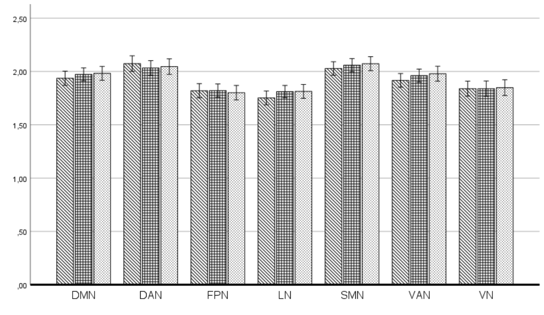

Fig. A.5. *mMSE* features (a. *AUC*, b. *MaxSlope*, c. *AvgEnt*) for particular resting-state networks across the timescales and three segments (bars marked with different textures: segment #1 (stripes), segment #2 (checkered), segment #3 (dotted)). DMN-default mode network, DAN-dorsal attention network, FPN-frontoparietal network, LN-limbic network, SMN-somatomotor network, VAN-ventral attention network, and VN-visual network.

A3.4. The changes in the entropy level across the timescales

The main effect of "Network" was significant (Greenhouse-Geisser-corrected $F(2.762, 259.674)=42.57$, $p<0.001$, $\eta^2_p=0.312$) (Fig. A.6).

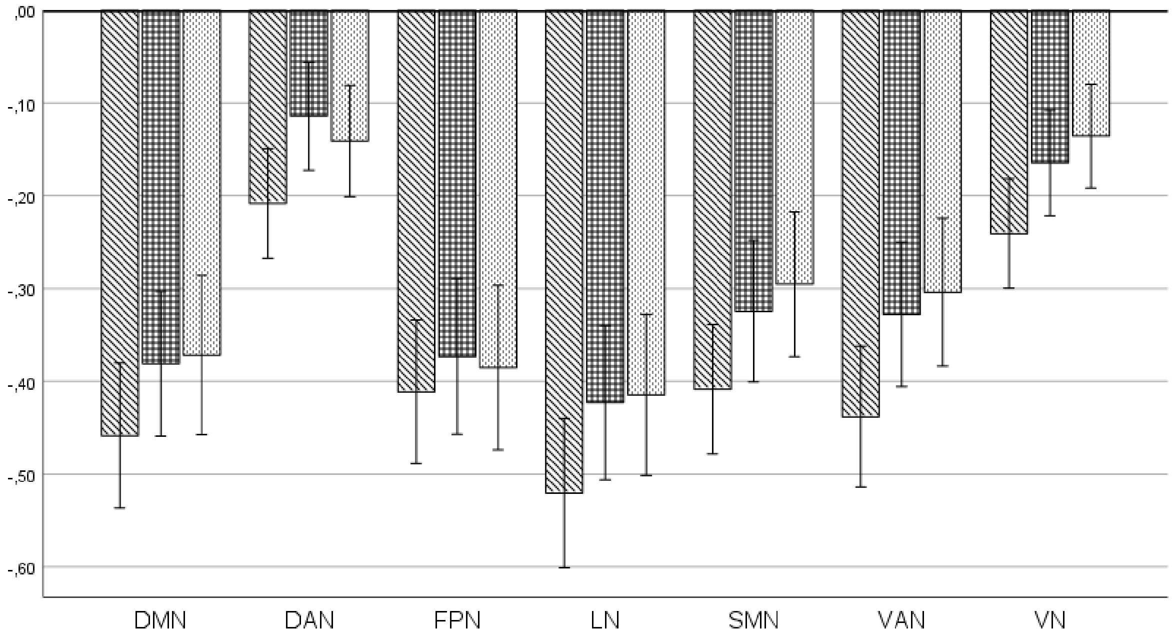

Fig. A.6. The dynamics of *MSE* changes (*DiffEnt:* the difference between #9 and #4 timescales) for particular resting-state networks across the timescales and three segments (bars marked with different textures: segment #1 (stripes), segment #2 (checkered), segment #3 (dotted)). DMN-default mode network, DAN-dorsal attention network, FPN-frontoparietal network, LN-limbic network, SMN-somatomotor network, VAN-ventral attention network, and VN-visual network. *DiffEnt* values for the limbic network were significantly higher compared to other networks. On the other hand, both DAN and VN exhibited significantly lower values.

A.3.4. Men and women are different in the rsEEG complexity at the fine and coarse timescales

For the *AUC*, the main effect of “S/g” (Greenhouse-Geisser-corrected F(1,92)=0.970, p=0.327, η^2_p=0.01), the “S/g × network” interaction (Greenhouse-Geisser-corrected F(3.36, 309.467)=0.910, p=0.446, η^2_p=0.01), “S/g × segment” interaction (Greenhouse-Geisser-corrected F(1.80, 165.645)=0.778, p=0.462, η^2_p=0.008), and the “S/g × network × segment” interaction (Greenhouse-Geisser-corrected F(4.32, 397.795)=0.852, p=0.50, η^2_p=0.01) were nonsignificant.

There was a significant main effect of “S/g” for the *MaxSlope* (Greenhouse-Geisser-corrected F(1,89)=4.30, p=0.04, η^2_p=0.046) and the *AvgEnt* (Greenhouse-Geisser-corrected F(1,92)=10.25, p<0.005, η^2_p=0.10). Women showed greater *MaxSlope* values (M=0.50±0.016) than men (M=0.455±0.015) whereas men had higher *AvgEnt* (M=2.006±0.034) than women (M=1.841±0.039). For both *MaxSlope* and *AvgEnt*, the “S/g × network” interaction was nonsignificant (*MaxSlope*: F(3.628, 322.927)=1.45, p=0.193, η^2_p=0.193, and *AvgEnt*: (F(3.35, 308.567)=0.611, p=0.627, η^2_p=0.007). The “S/g × segment” interaction (*MaxSlope*: F(1.66, 148.084)=1.049, p=0.354, η^2_p=0.012, and *AvgEnt*: (F(1.803, 156.87)=0.01, p=0.986, η^2_p=0.001), and the “S/g × segment × network” (*MaxSlope*: F(9.25, 822.958)=1.532, p=0.86, η^2_p=0.006, and *AvgEnt*: (F(4.883, 449.246)=0.873, p=0.497, η^2_p=0.009) interaction were either nonsignificant .

We found a significant main effect of “S/g” for the *DiffEnt* (Greenhouse-Geisser-corrected F(1,92)=10.128, p<0.005, η^2_p=0.10). In general, women showed greater differences between the #9 and #4 scales (M=-0.431±0.055) than men (M =-0.221±0.044). The “S/g × network” (Greenhouse-Geisser-corrected F(2.447, 225.14)=2.077, p=0.117, η^2_p=0.022) and “S/g × network × segment” (Greenhouse-Geisser-corrected F(8.37, 770.25)=1.28, p=0.246, η^2_p=0.014) interaction was non-significant.

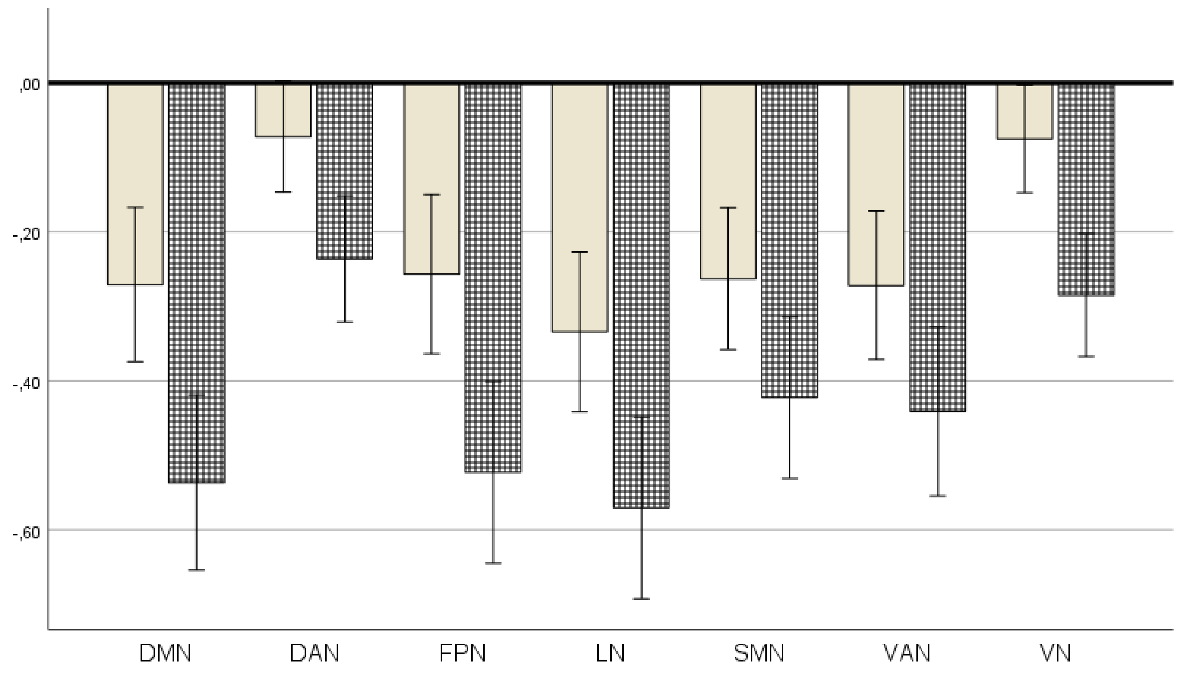

Fig. A.7. The s/g-related differences in the dynamics of MSE changes (*DiffEnt*: the difference between #9 and #4 timescales) for particular resting-state networks across the timescales. DMN-default mode network, DAN-dorsal attention network, FPN-frontoparietal network, LN-limbic network, SMN-somatomotor network, VAN-ventral attention network, VN-visual network. Males - bars marked with beige; females - bars marked with checkered texture.
